# A Flow Cytometry Method for Dissecting the Cell Differentiation Process of *Entamoeba* Encystation

**DOI:** 10.3389/fcimb.2018.00250

**Published:** 2018-07-24

**Authors:** Fumika Mi-ichi, Yasunobu Miyake, Vo Kha Tam, Hiroki Yoshida

**Affiliations:** Division of Molecular and Cellular Immunoscience, Department of Biomolecular Sciences, Faculty of Medicine, Saga University, Saga, Japan

**Keywords:** flow cytometry, *Entamoeba*, encystation, cell differentiation, amoebiasis

## Abstract

Amoebiasis is caused by *Entamoeba histolytica* infection, a protozoan parasite belonging to the phylum Amoebozoa. This parasite undergoes a fundamental cell differentiation process from proliferative trophozoite to dormant cyst, termed “encystation.” The cysts formed by encystation are solely responsible for the transmission of amoebiasis; therefore, *Entamoeba* encystation is an important subject from both biological and medical perspectives. Here, we have established a flow cytometry strategy for not only determining the percentage of formed cysts but also for monitoring changes in cell populations during encystation. This strategy together with fluorescence microscopy enables visualization of the cell differentiation process of *Entamoeba* encystation. We also standardized another flow cytometry protocol for counting live trophozoites. These two different flow cytometry techniques could be integrated into 96-well plate-based bioassays for monitoring the processes of cyst formation and trophozoite proliferation, which are crucial to maintain the *Entamoeba* life cycle. The combined two systems enabled us to screen a chemical library, the Pathogen Box of the Medicine for Malaria Venture, to obtain compounds that inhibit either the formation of cysts or the proliferation of trophozoites, or both. This is a prerequisite for the development of new drugs against amoebiasis, a global public health problem. Collectively, the two different 96-well plate-based *Entamoeba* bioassay and flow cytometry analysis systems (cyst formation and trophozoite proliferation) provide a methodology that can not only overcome the limitations of standard microscopic counting but also is effective in applied as well as basic *Entamoeba* biology.

## Introduction

*Entamoeba histolytica* is the causative agent of amoebiasis. Amoebiasis is a global public health problem owing to its high morbidity and mortality rates (Ralston and Petri, 2011; Watanabe and Petri, 2015). High numbers of individuals are infected with *E. histolytica* but are asymptomatic and do not require treatment. However, they are important because they unconsciously spread the disease (Watanabe and Petri, 2015). Clinical treatment is currently inadequate because only a few drugs are available, and an effective vaccine has not been developed (Haque et al., 2003; Quach et al., 2014).

*E. histolytica*, a protozoan parasite belonging to the phylum Amoebozoa, survives drastic environmental changes outside as well as inside its human host, by alternating its form between proliferative trophozoite and dormant cyst. These two distinct stages are connected by two cell differentiation processes: “encystation” and “excystation.” Encystation is a process for differentiation of trophozoite into cyst whereas excystation is that of cyst into trophozoite. Trophozoites colonize the large intestine and proliferate there. Some of them differentiate into cysts. These cysts are excreted, and are then ingested by new hosts and reach the small intestine, where they hatch into trophozoites (Watanabe and Petri, 2015; Mi-ichi et al., 2016). Encystation, a parasitic strategy involving a fundamental cell differentiation process, appears simple but is closely associated with transmission of the disease. The transmission of amoebiasis is solely mediated by cysts formed by encystation; therefore, inhibition of encystation is an effective strategy against amoebiasis. Hence, *Entamoeba* encystation is an important subject from a medical as well as a biological perspective. Nevertheless, the underlying molecular and cellular mechanisms require further elucidation (Mi-ichi et al., 2016).

In this study, we describe a method for counting *Entamoeba* cysts, an indispensable procedure in *Entamoeba* encystation studies. It is a flow cytometry method using premixed calcofluor (CF) and Evans blue (EB) dyes, which is rapid and quantitative, providing reproducible and reliable data. By exploiting this method together with fluorescence microscopy, we visualized differentiating cells that appeared during *Entamoeba* encystation. We also standardized a flow cytometric protocol to separately count live and dead *E. histolytica* trophozoites. By combining these two different 96-well plate-based systems we were able to screen a chemical library for potential leads that inhibit *Entamoeba* encystation and/or trophozoite proliferation, which is a prerequisite step for the development of new drugs against amoebiasis. To confirm the effectiveness of this combined system, we screened 400 compounds exhibiting diverse scaffolds from the Pathogen Box of the Medicine for Malaria Venture (MMV; https://www.pathogenbox.org/).

## Materials and methods

### Chemicals

Calcofluor White Stain, a premixed CF (Fluorescent Brightener 28) and EB dye, was purchased from Sigma-Aldrich (St. Louis, Mo, USA). CF and EB were from Sigma-Aldrich and Nacalai Tesque (Kyoto, Japan), respectively. *N*-Lauroylsarcosine sodium salt (>94.0% purity) was from Sigma-Aldrich.

Lactacystin (>94.2% purity) and polyoxin D (>94.5% purity) were purchased from Biolinks Co. Ltd. (Tokyo, Japan) and Kaken Pharmaceutical Co. Ltd. (Tokyo, Japan), respectively, whereas metronidazole (>98.0% purity) and paromomycin (>98.0% purity) were both from Sigma-Aldrich. All four compounds were dissolved in sterilized water, respectively, at 1, 10, 50, and 50 mM as the stock solutions. Aliquots of 50 μL were stored at −30°C, and freeze-thaw cycles were less than two before use.

The Pathogen Box, 400 compounds exhibiting diverse scaffolds, was provided by MMV (https://www.pathogenbox.org/); each compound was dissolved in DMSO at 10 mM and distributed into individual wells of 96-well plates (10 μL/compound and 80 compounds/plate). Ninety microliters of DMSO were then added to each well and then 10 replicates were made (10 μL aliquots of all the compounds' stocks at 1 mM) and stored at −30°C. When needed, a set of replicates covering all 400 compounds (10 μL aliquoted at 1 mM each) was thawed, and 1 μL for the trophozoite proliferation assay and 2.4 μL for the cyst formation assay was dispensed into wells of a 96-well culture plate to make a replicate. Auranofin (>98% purity) (which is identified as E-H-05 in the Pathogen Box) was also purchased from Sigma-Aldrich, dissolved in DMSO to 10 mM and dispensed into 50 μL aliquots for storage at −30°C.

### Parasite culture and sample preparations for each analysis

*E. invadens* (IP-1) and *E. histolytica* (HM-1:IMSS cl6) were routinely maintained as described (Mi-ichi et al., 2009, 2015). For the cyst formation assay using *E. invadens*, encystation inducing treatment was performed as described (Mi-ichi et al., 2015), except that *E. invadens* trophozoites suspended in encystation medium were seeded in a 96-well culture plate (240 μL per well) and the plate was sealed as described (Suresh et al., 2016) using Parafilm® from Bemis Flexible Packaging (Neenah, WI, USA). Note that the final cell density of 6 × 10^5^ cells/mL was not different from that in Mi-ichi et al. (2015); therefore, the initial number of cells per well was 1.44 x 10^5^. After incubating at 26°C for the period indicated, cells in 96-well culture plates were harvested by centrifugation at 440 × g for 5 min at 4°C. Cell pellets were then suspended in 120 μL PBS containing an appropriate staining reagent, a premixed CF and EB, CF, or EB. The premixed CF and EB, Calcofluor White Stain, was diluted 5-fold with PBS just before use; final concentrations of CF and EB used were 0.2 and 0.1 mg/mL, respectively. The working solutions of CF and EB were prepared just before use by 5-fold dilution of the stocks with PBS to 0.2 and 0.1 mg/mL, respectively, and stored at room temperature. The obtained cell suspensions were held for 15 min at room temperature and then precipitated by centrifugation at 440 × g for 5 min at 4°C, washed with 120 μL PBS, and precipitated again. Finally, the cell pellet was resuspended in flow cytometry buffer (0.5% BSA, 2 mM EDTA, and 0.05% azide in PBS) for injection into a flow cytometer [MACSQuant from Miltenyi Biotec (Bergisch Gladbach, Germany)]. When needed, sarcosyl treatment was performed before the staining step, as described previously (De Cádiz et al., 2013; Mi-ichi et al., 2015).

For the *E. histolytica* trophozoite proliferation assay, trophozoites were harvested from a routine culture by centrifugation at 440 × g for 5 min at 4°C, and the harvested cells were resuspended in fresh standard culture medium (Mi-ichi et al., 2009). A 96-well culture plate was then seeded with the obtained cell suspension (100 μL per well; final cell density, 1 × 10^5^ cells/mL); therefore, the initial number of cells per well was 1 x 10^4^. After incubation at 37°C for 24 h under anaerobic conditions using Anaerocult A (Merck), cells in the 96-well culture plate were harvested, stained with 1.0 μg/mL PI and processed for flow cytometry analysis as described above for *E. invadens*.

For the treatment of *E. invadens* or *E. histolytica* with compounds, each stock solution of lactacystin, polyoxin D, metronidazole, paromomycin, or auranofin was serially diluted in the medium used for each treatment. As controls, water was used in place of the first four compound solutions and the final water content in a well was 1% (vol/vol) whereas DMSO was used as the control of auranofin and the final DMSO content was 1% (vol/vol). The highest control solvent content in any well was 1% (vol/vol). Each compound was added when the cells were seeded into 96-well culture plates and incubated for 72 h in the cyst formation assay or for 24 h in the trophozoite proliferation assay. When needed, compounds were added 48 h after induction of encystation and cells then analyzed by a flow cytometer as described above.

For screening the 400 Pathogen Box compounds, 1 mM of each compound stock solution or DMSO control were added to the culture medium for each assay at 1% (vol/vol).

### Conditions for flow cytometry analysis

A MACSQuant from Miltenyi Biotec was used as a 96-well plate-based flow cytometer. CF was excited using a 405 nm laser, and the fluorescence emission was collected using a 450/50 filter. EB and PI were both excited using a 488 nm laser and the fluorescence emission was collected using a 614/50 nm filter (Hed et al., 1983). The processing volume was set at 30 μL from the 120 μL fluorescent dye(s)-treated cell suspension in each well prepared as described above. The obtained data were analyzed using Flow Jo software (Tree Star, Ashland, OR, USA).

After the samples from either an encystation-inducing culture or a standard culture for trophozoite proliferation were treated with staining solutions, the flow cytometry analysis of the prepared samples was completed within 3 or 1 h, respectively; the stability of the prepared samples was confirmed by the reproducibility of data from the chemical library screening. It should be mentioned that samples prepared for the trophozoite proliferation assay were suspended by pipetting just before processing in the flow cytometer.

### Fluorescence microscopy

A portion of the *E. invadens* sample prepared for flow cytometry analysis, as described above, was examined under a BZ-9000 fluorescence microscope (Keyence, Osaka, Japan). The obtained images were processed using BZ-II software (Keyence).

## Results

### Establishing a flow cytometry method for measuring *Entamoeba* cyst numbers

In *Entamoeba* encystation studies, the *in vitro* culture of *E. invadens*, a reptilian parasite, and not that of *E. histolytica*, has been adopted as a model system. This is mainly because laboratory strains of *E. histolytica* do not encyst after adaptation to *in vitro* culture conditions; however, *E. invadens* strains are able to undergo *in vitro* encystation (Sanchez et al., 1994; Coppi and Eichinger, 1999; Mi-ichi et al., 2016). *In vitro* encystation is usually induced by transfer of proliferating trophozoites from a standard culture to encystation conditions (Sanchez et al., 1994; Mi-ichi et al., 2015). In encystation-inducing conditions, the number of cysts formed increases with the length of incubation and then reaches a plateau. In a standard encystation assay, measurement is manually performed under a microscope at 72 h post induction (Sanchez et al., 1994).

**Figure 1:**
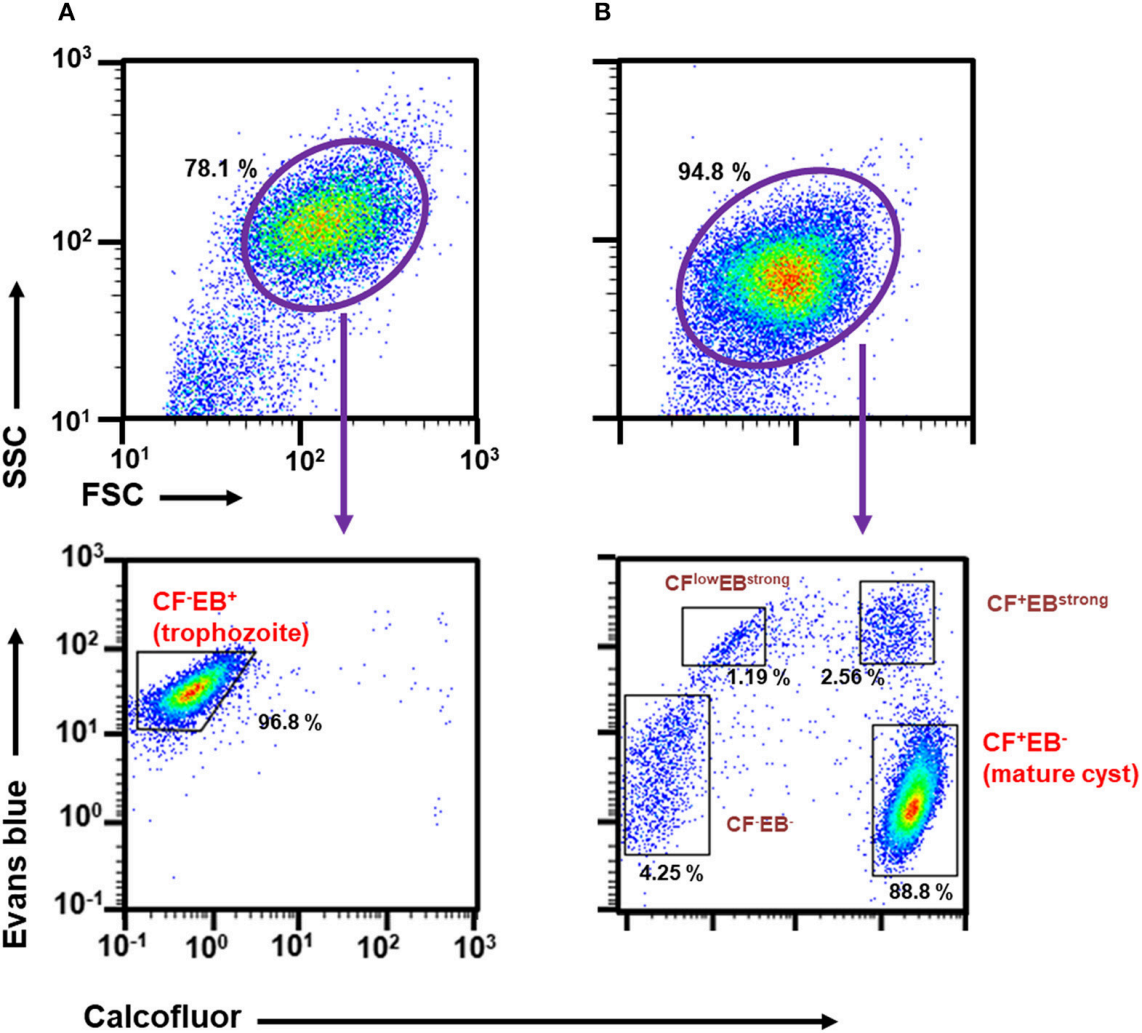
Characterization of *Entamoeba* cells in routine culture and in the encystation-inducing culture at 72 h after induction by flow cytometric analysis. **(A)** *E. invadens* trophozoites prepared from the routine culture. **(B)** *E. invadens* cells from the encystation-inducing culture at 72 h after induction. Representative data are shown from three independent experiments.

To obtain accurate data, reproducible distinction between trophozoites and cysts is essential. Flow cytometry has been recently reported as a suitable method for this (Welter et al., 2017). Here, we attempted to introduce and standardize conditions to achieve these criteria. Samples prepared from routine trophozoite proliferation cultures gave a single population by forward scatter (FSC)/side scatter (SSC) analysis (Figure 1A, upper panel). Samples prepared from encystation-inducing cultures at 72 h after induction also only gave a single population, although its area was moved to a slightly lower FSC/SSC position (Figure 1B, upper panel).

**Figure 2:**
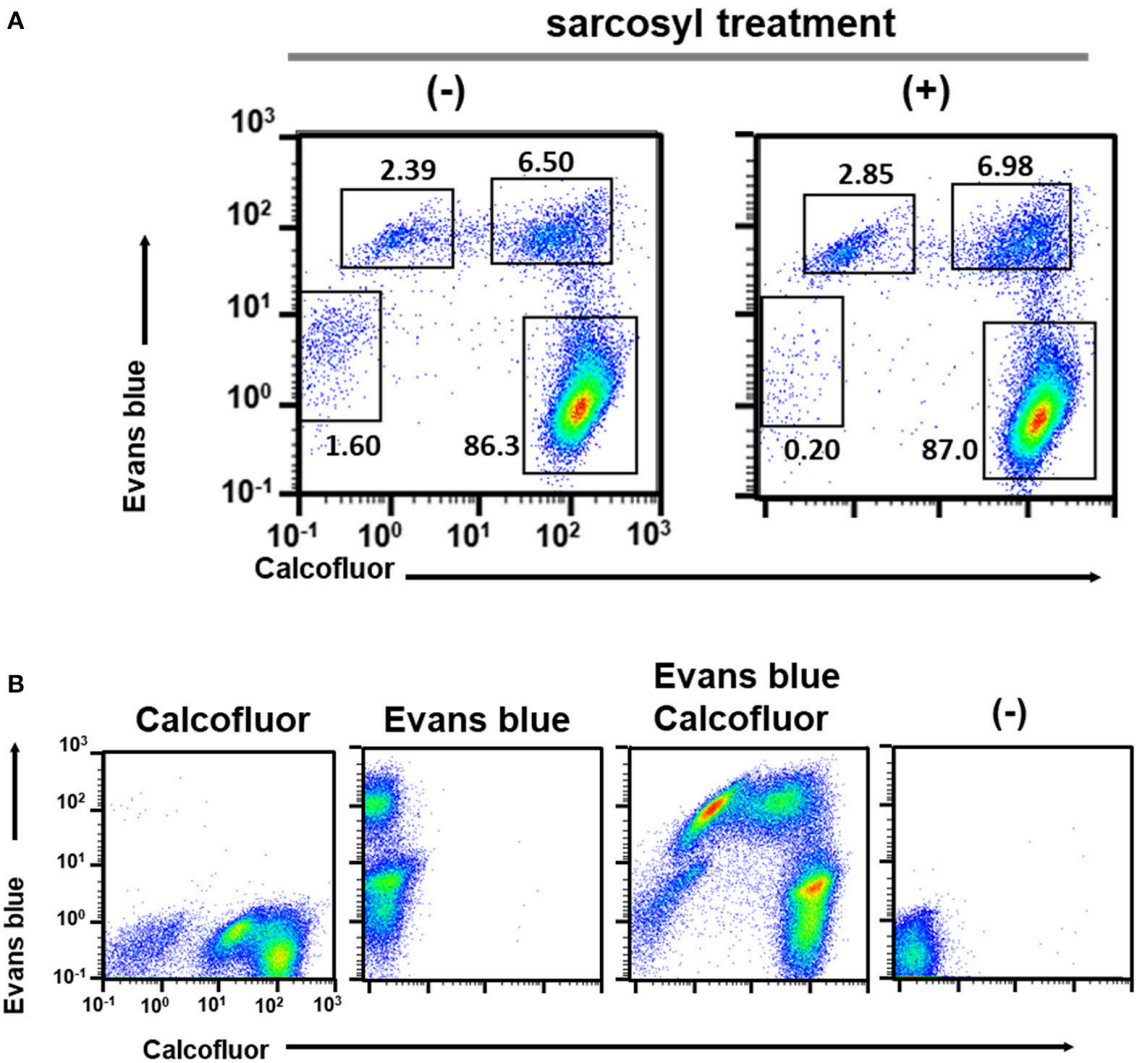
Characterization of cell populations in the encystation-inducing culture at 72 h after induction by flow cytometric analysis. **(A)** Sarcosyl-treatment. **(B)** Single and double staining using CF and EB dyes.

We then focused on CF and EB dyes to differentially stain trophozoites and cysts. CF is a fluorochrome that binds to structures containing chitin, a component in the *Entamoeba* cyst wall, and has, therefore, been used in standard microscopy methods (Arroyo-Begovich et al., 1980; Herrera-Martínez et al., 2013). EB was tentatively used for staining live *Entamoeba* cells because it was shown to stain the plasma membrane of viable human neutrophils (Hed et al., 1983). To confirm that these two dyes can differentiate between trophozoites and cysts, trophozoites in a routine culture and cells in an encystation-inducing culture at 72 h after induction were separately stained with a premixed reagent of CF and EB dyes, Calcofluor White Stain (Sigma-Aldrich) (Figures 1A,B, lower panels). As expected, the single FSC/SSC population detected in the trophozoite culture gave only a single CF negative (CF^−^) and EB positive (EB^+^) population, indicating that the trophozoites can be isolated as a CF^−^/EB^+^ population (Figure 1A, lower panel). In contrast, the encystation-inducing culture consisted of several distinct populations. The largest population exhibited CF and not EB fluorescence (CF^+^/EB^−^). Another two populations also produced CF fluorescence and stronger EB fluorescence than trophozoites (CF^low^/EB^strong^ and CF^+^/EB^strong^) (Figure 1B, lower panel). Most cells in these three populations remained after sarcosyl treatment, which is used to eliminate trophozoites to facilitate the counting of cysts (Figure 2A; De Cádiz et al., 2013). Additionally, one population of cells exhibiting neither CF nor EB fluorescence, was detected (CF^−^/EB^−^) (Figure 1B, lower panel). This population partly overlapped with the CF^−^/EB^+^ population, which consists of trophozoites (see Figure 1A, lower panel). Furthermore, the cells in this population were not resistant to sarcosyl treatment (Figure 2A).

**Figure 3:**
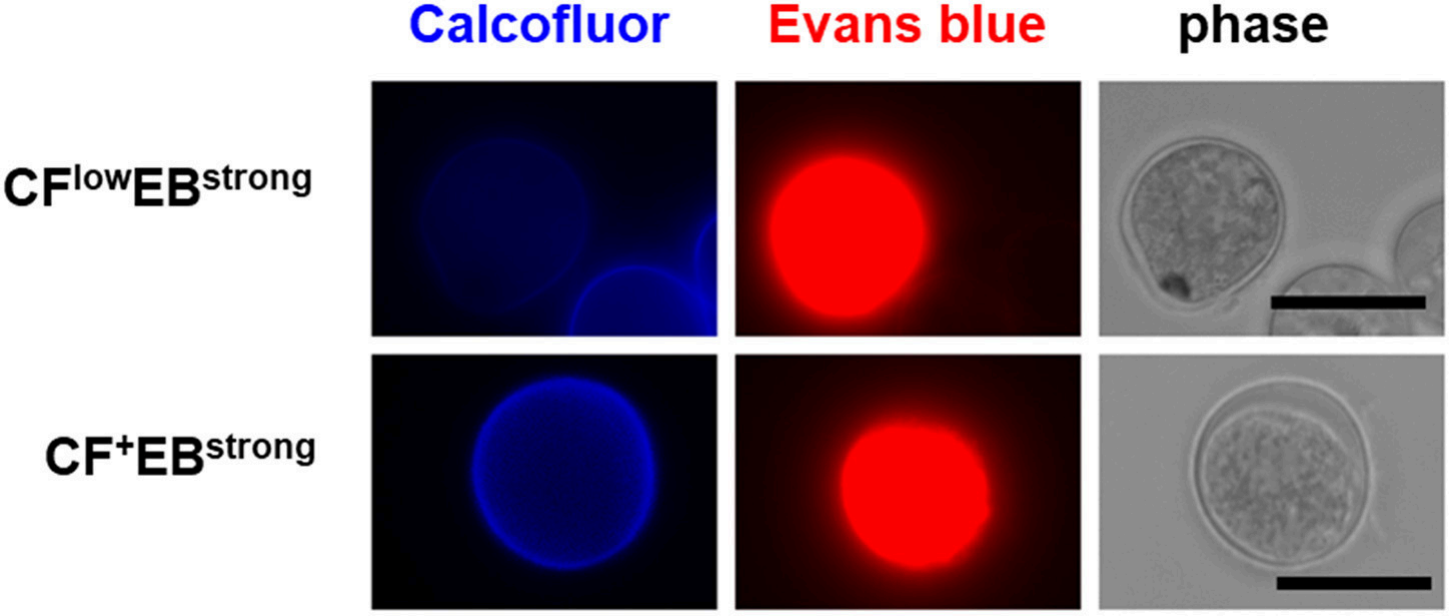
Fluorescence microscopy images of the *Entamoeba* cells observed as the minor population in the encystation-inducing culture at 72 h after induction. Bar indicates 10 μm. Representative images are shown from two independent experiments.

All five populations (CF^−^/EB^+^ in the trophozoite culture and CF^+^/EB^−^, CF^low^/EB^strong^, CF^+^/EB^strong^, and CF^−^/EB^−^ in the encystation-inducing culture) can be detected in a fluorescent reagent-binding dependent manner. Staining with either CF or EB alone provided cell distributions along only the CF- or EB-signal axis, respectively (Figure 2B). Under a fluorescence microscope, the most frequently observed cells in encystation-inducing culture at 72 h post induction gave CF and almost no EB fluorescence, consistent with the flow cytometry results. Furthermore, these cells were round and surrounded by a thick layer (see 72 h images in **Figure 5**). Round cells yielding EB as well as CF fluorescence, or yielding EB and almost no or no CF fluorescence were also be observed at much lower frequencies (Figure 3). Collectively, these results indicate that the majority of cells in the *Entamoeba* encystation-inducing culture at 72 h after induction are round, surrounded by a thick chitin cyst wall to which CF dye binds and are resistant to sarcosyl treatment, and emit CF and not EB fluorescence; in other words, based on their morphology, composition and properties against detergent treatment, the major CF^+^/EB^−^ population consists of cells showing typical features of mature *Entamoeba* cysts.

Consistency of mature *Entamoeba* cyst counting obtained by the above flow cytometry analysis and standard microscopy counting was confirmed using an encystation-inducing culture at 72 h after induction. For instance, the flow cytometry analysis calculated mature cyst densities to be 4.47 × 10^5^ cells/ml, whereas the microscopy method performed by counting at most a few hundred cells as described by (Mi-ichi et al., 2015) gave densities of 4.80 × 10^5^ cells/ml.

Hence, these results demonstrate a method for counting mature *Entamoeba* cysts, which is rapid and quantitative, providing reproducible and reliable data. Furthermore, the method can be integrated into a 96-well plate-based *Entamoeba* encystation bioassay using flow cytometry and premixed CF and EB.

### Monitoring the cell differentiation process of *Entamoeba* encystation

Our 96-well plate-based *Entamoeba* bioassay can count the number of mature cysts formed by encystation (see the above section). This system is an alternative to the standard microscopy method used in the *Entamoeba* field. Moreover, from the results demonstrated in the above section, we predict that this methodology will also be effective in studying the molecular and cellular mechanisms underlying encystation. To confirm our prediction, we monitored the dynamics of population changes during the course of encystation by characterization of populations using flow cytometry analysis in combination with fluorescence microscopy.

**Figure 4:**
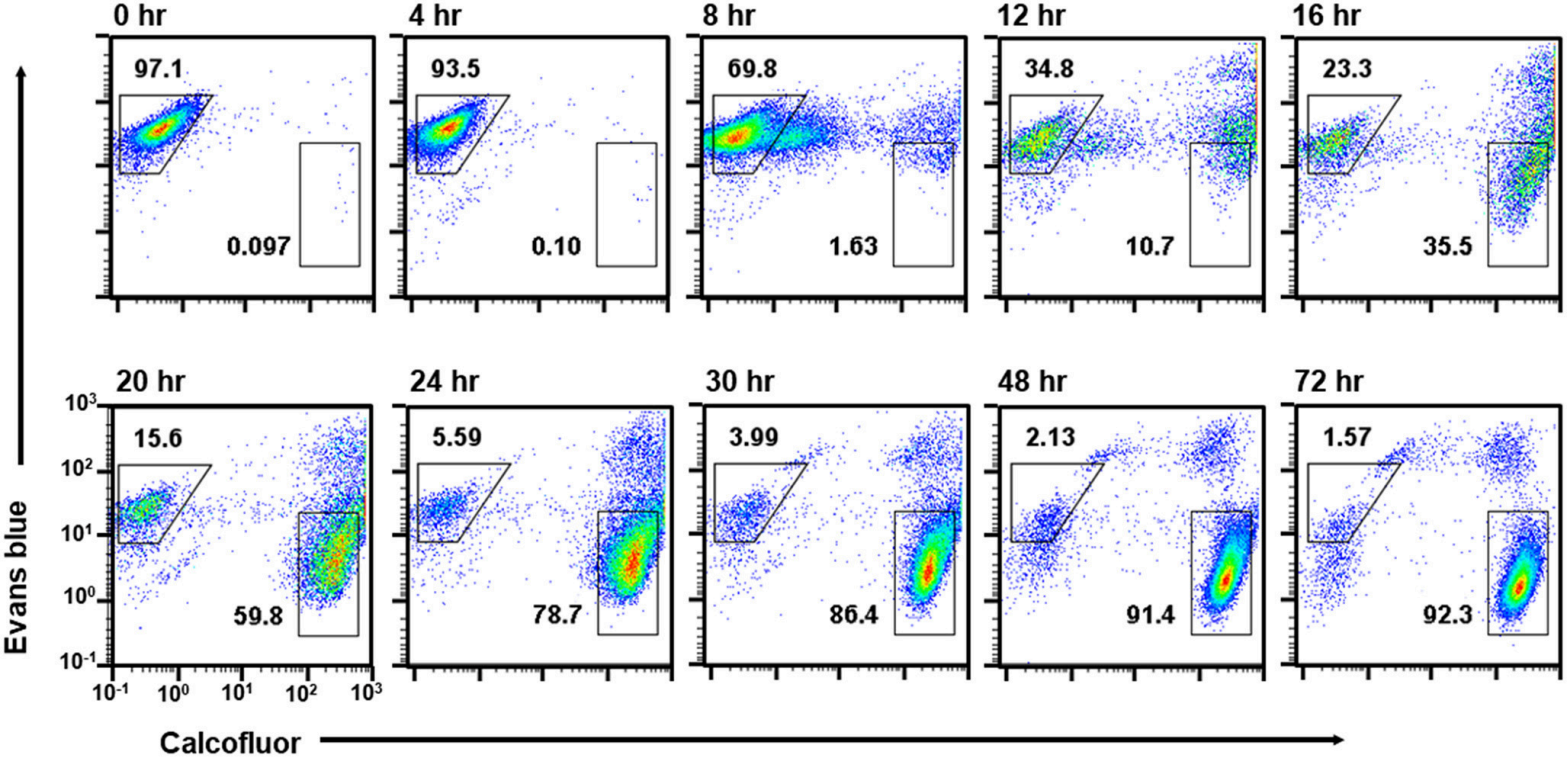
Time course analysis of cell populations that appeared during *Entamoeba* encystation. *E. invadens* cultures induced for encystation were analyzed at the indicated time after induction. Representative data are shown from two independent experiments.

**Figure 5:**
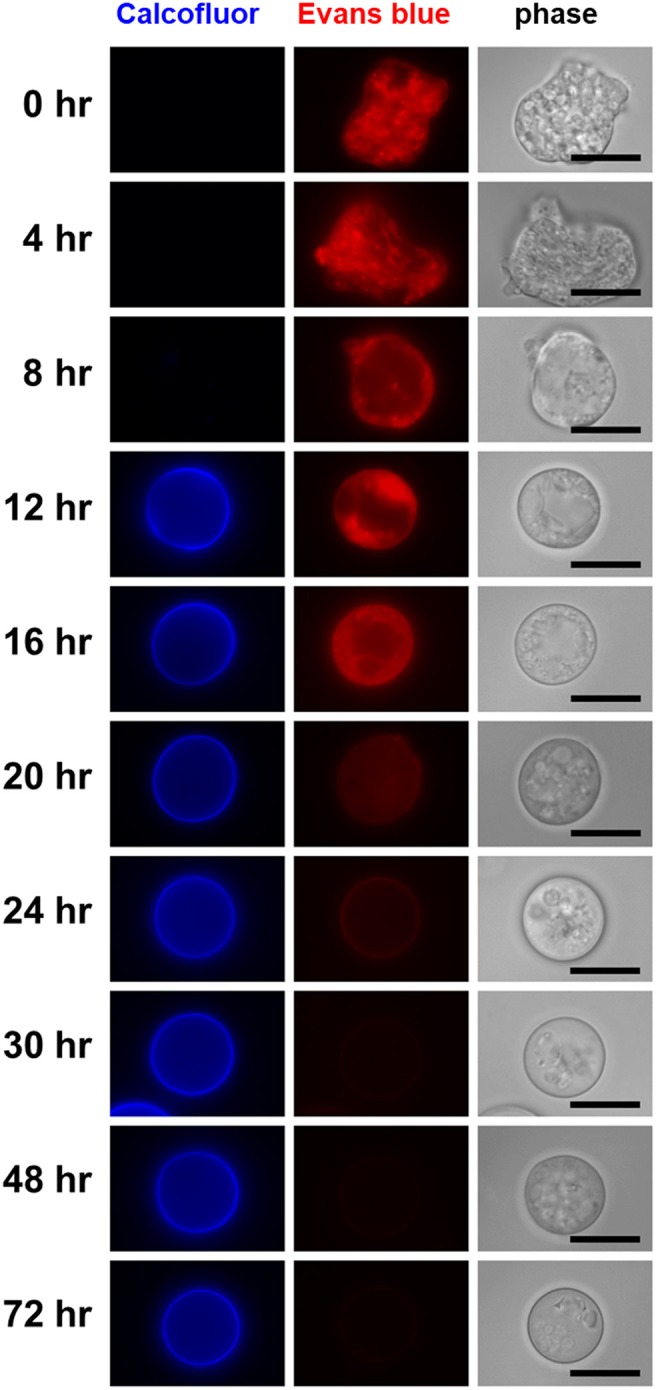
Visualization of differentiating cells that appeared during the course of *Entamoeba* encystation. In the encystation-inducing culture, cells observed as the dominant population at the indicated times after induction are shown by fluorescence microscopy. Bar indicates 10 μm. Representative images are demonstrated from two independent experiments.

The only population detected at 0 h was the single CF^−^/EB^+^ population, which is composed of proliferating trophozoites (Figure 4; see Figure 1A, lower panel). The cells giving EB- and not CF-signal were confirmed to have trophozoite morphology by fluorescence microscopy (Figure 5). Similar results were obtained by both flow cytometry analysis and fluorescence microscopy at 4 h after induction of encystation (Figures 4, 5).

The single CF^−^/EB^+^ population detected up to 4 h after induction, then became smaller and smaller from 8 h on (Figure 4). Inversely, two new populations, CF^low^/EB^+^ and CF^+^/EB^+^ were generated (Figure 4). Consistent with the flow cytometry results, fluorescence microscopy showed that from 8 h, CF fluorescent cells became evident, while cells giving almost no CF fluorescence were also still detected, and their sizes and morphologies were smaller and less motile than those of proliferating trophozoites. From 12 h, the most frequently observed cells showed very similar size and morphology to the mature cyst, but unlike mature cysts, both gave EB and CF fluorescence (Figure 5).

The CF^+^/EB^+^ population moved lower and lower along the EB signal axis from 12 h and reached a position similar to that of the mature cyst population (CF^+^/EB^−^) at 72 h (Figure 4; see Figure 1B, lower panel and the 4th paragraph in the 1st section). Consistently, fluorescence microscopy revealed that cells mainly observed from 12 to 16 h were round and gave EB as well as CF fluorescence, but, from 16 h, cells lost the EB but retained the CF signal and the CF^+^/EB^−^ population became dominant from 30 h (Figure 5). These results indicate that the CF^+^/EB^+^ population sequentially becomes the CF^+^/EB^−^, or mature cyst population.

**Figure 6:**
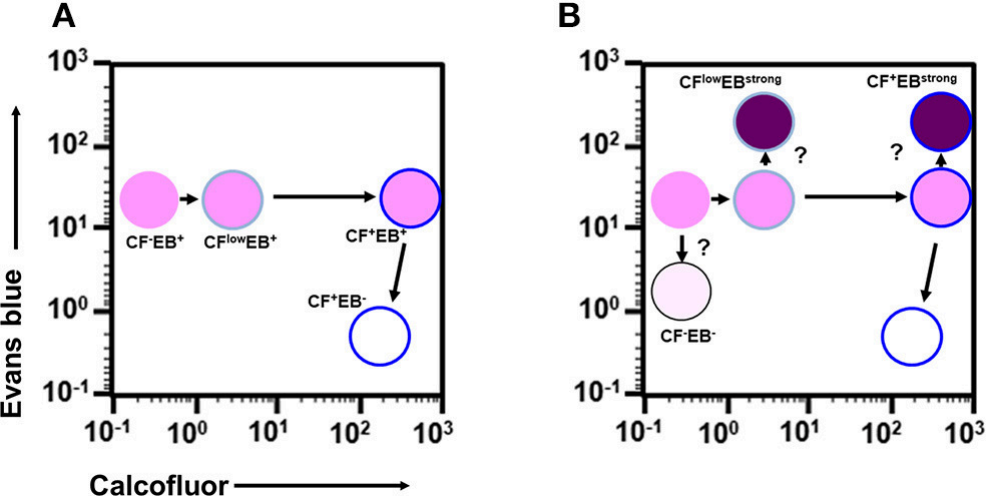
Proposed scenario for the cell differentiation process of *Entamoeba* encystation. **(A)** Major route from proliferative trophozoite to dormant, mature cyst via two precursor cells. **(B)** Relationship among all cells that appeared during encystation.

Taken together, analysis of the time course enabled visualization of the main differentiation process of *Entamoeba* encystation: proliferating trophozoites (CF^−^/EB^+^ population) become dormant cysts (CF^+^/EB^−^ population) via CF^low^/EB^+^ and CF^+^/EB^+^ populations. Furthermore, it showed that cells in the CF^+^/EB^+^ population were already becoming round and surrounded by chitin and that their cellular content had reached a plateau. These cells were losing permeability to solutes, such as EB, probably because they were acquiring a complete cyst wall structure like the mature cysts in the CF^+^/EB^−^ population (Figure 6A).

### Effectiveness of combining the two 96-well plate-based *Entamoeba* bioassay and flow cytometry systems for the development of new drugs against amoebiasis

#### Assessment of the 96-well plate-based *E. invadens* encystation assay connected to flow cytometry for chemical library screening

To develop new preventive measures against amoebiasis, such as anti-amoebic and amoebiasis transmission-blocking drugs, screening potential leads from chemical libraries is a prerequisite toward the ultimate goal. To assess the applicability of the above system, the *E. invadens* encystation bioassay was integrated with flow cytometry and the model compounds, lactacystin, and polyoxin D, which show a significant inhibitory effect on encystation (Avron et al., 1982; Gonzalez et al., 1999), were assayed. Metronidazole and paromomycin, which are clinically used to treat amoebiasis patients, were also assayed (Marie and Petri, 2013; Penuliar et al., 2015). In the present assay system, the inhibitory effect of different compounds on encystation was evaluated by the encystation rate expressed as the mature cyst population percentage (CF^+^/EB^−^) in each sample relative to that in a solvent-treated control (set as 100%).

**Figure 7:**
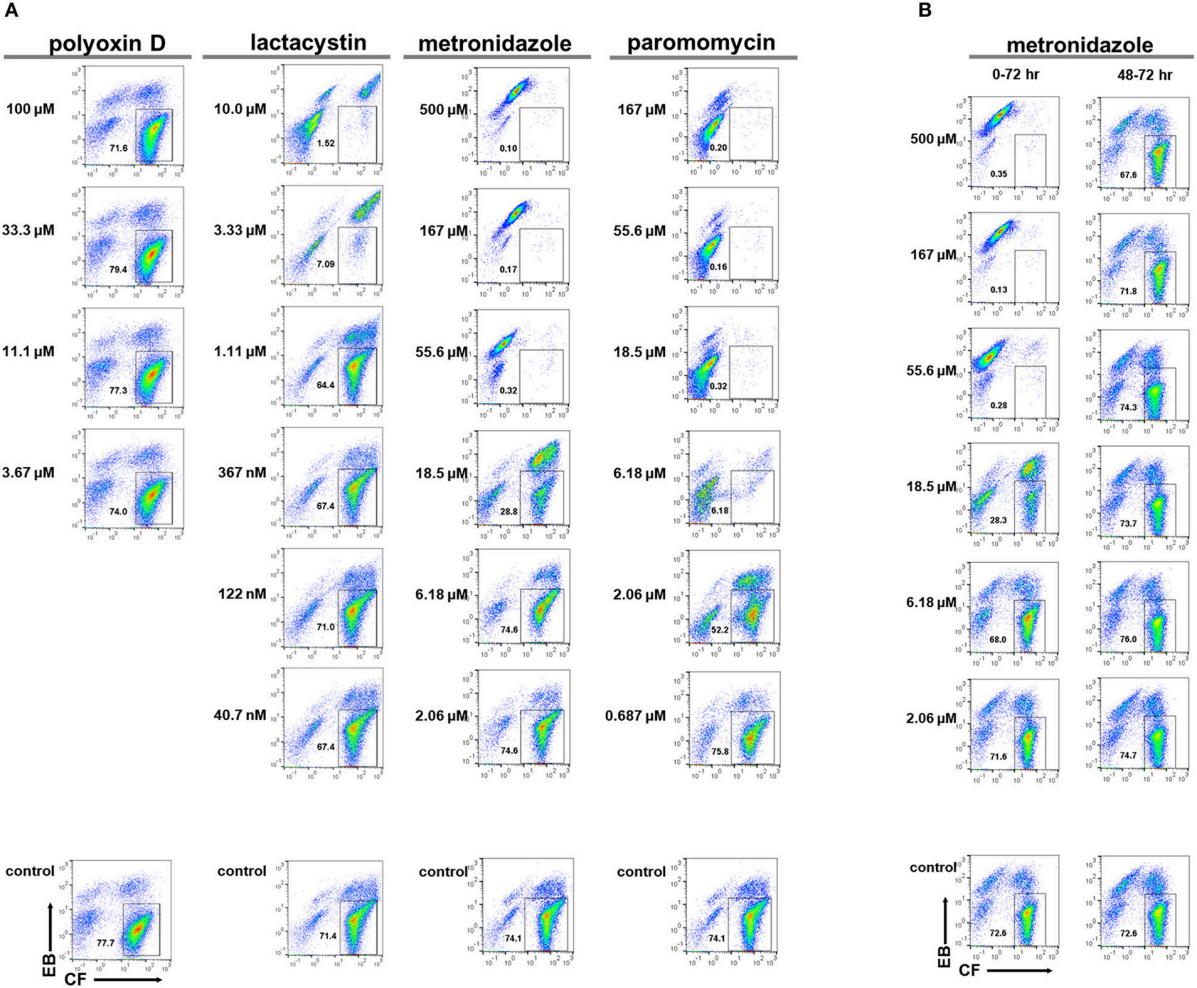
Effects of model compounds, lactacystin, polyoxin D, metronidazole, and paromomycin, on *E. invadens* cyst formation. **(A)** Encystation-inducing culture was treated with each compound immediately after induction (0–72 h). **(B)** Encystation-inducing culture was treated with metronidazole 48 h after induction. Flow cytometric analysis was performed 72 h after induction (48–72 h). Representative data are shown from three experiments in **(A,B)**, respectively.

Lactacystin inhibited cyst formation (reduction of the CF^+^/EB^−^ population) with an IC_50_ value of 1.56 ± 0.251 μM, while polyoxin D did not show any inhibitory effect (Figure 7A). The IC_50_ value of lactacystin was close to the previously reported value of 1.25–2.5 μM (Gonzalez et al., 1999). However, the result for polyoxin D was inconsistent with one previous study, which showed a dose-dependent inhibition of cyst formation at 2–500 μg/mL (Avron et al., 1982), but was supported by another study that demonstrated no effect on chitin synthase activity, a predicted target of polyoxin D, in cyst lysate at 100 μg/mL (Das and Gillin, 1991). Furthermore, at ~IC_99_, lactacystin caused significant accumulation of the CF^−^/EB^−^ population (Figure 7A, 10 μM).

Metronidazole and paromomycin also inhibited cyst formation with IC_50_ values of 14.7 ± 3.06 and 2.65 ± 0.311 μM, respectively (Figure 7A). At ~IC_99_s, they also caused irregularities in population distribution; metronidazole caused significant accumulation of the CF^low^/EB^strong^ population (Figure 7A, 55.6 μM) whereas paromomycin caused significant accumulation of the CF^−^/EB^−^ and CF^low^/EB^strong^ populations (Figure 7A, 18.5 μM). These results suggest that both metronidazole and paromomycin, similar to lactacystin, possess inhibitory activity against encystation. Alternatively, the possibility exists that all three compounds affect only trophozoites in both culture conditions used for trophozoite proliferation and cyst formation assays; in the encystation-inducing culture, there is a lag time to commit to trophozoite differentiation.

Metronidazole was added to *Entamoeba* encystation-inducing cultures at 48 h post induction at six different concentrations ranging from 2.06 to 500 μM. Samples were then analyzed at 72 h post induction. None of the concentrations tested, even 500 μM [~10 times higher than the IC_99_ concentration (see Figure 7A)], affected the number of cysts formed at 72 h compared with the present encystation assay (0–72 h) (Figure 7B). Flow cytometry and fluorescence microscopy indicated that the majority of cells in the culture at 48 h post induction were similar to mature cysts (see Figures 4, 5). Collectively, these results indicate that the majority of *Entamoeba* cells in the encystation-inducing culture at 48 h post induction were tolerant to metronidazole. This finding indicates that the halting of cyst formation by metronidazole observed in the present encystation assay (0–72 h) is not a direct effect on cells that show similar characteristics to mature *Entamoeba* cysts.

#### Standardizing the 96-well plate-based flow cytometry to count live *E. histolytica* trophozoites, which can be integrated into the proliferation assay

**Figure 8:**
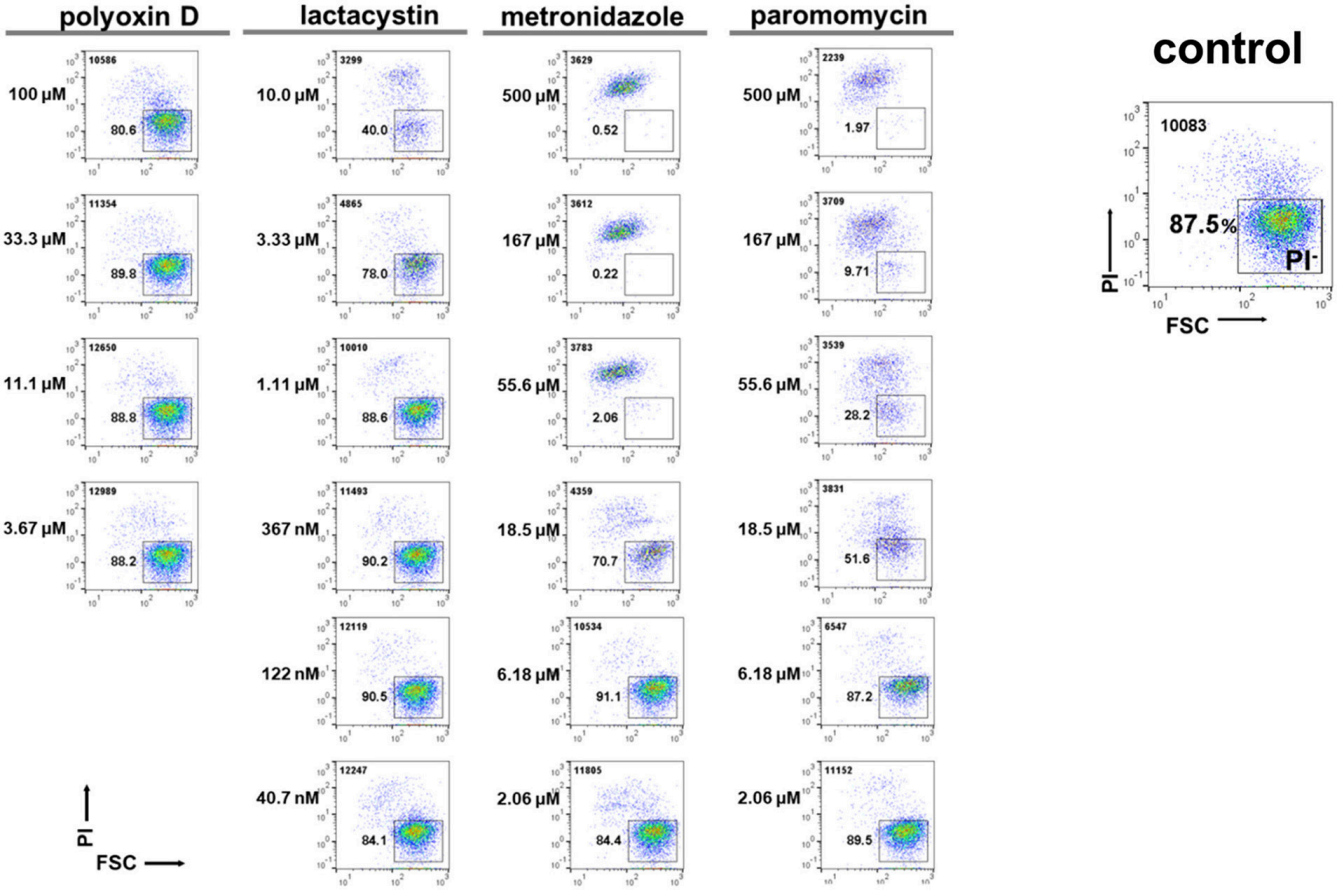
Effects of model compounds, lactacystin, polyoxin D, metronidazole, and paromomycin, on *E. histolytica* trophozoite proliferation. The total cell number counted is indicated in the upper left corner. Representative data are shown from three experiments.

Whether a compound identified in the encystation screen exerts an effect on trophozoite growth is among the most important questions that should be addressed to further characterize the compound. For this assessment, we attempted to standardize the flow cytometry analysis using propidium iodide (PI), a membrane-impermeable dye that specifically stains dying or dead cells (Chatterjee et al., 2015). In the established assay system, the inhibitory effect of different compounds on trophozoite proliferation is evaluated by the live cell rate, which is calculated using the live trophozoite cell number [PI negative (PI^−^) population] and the total cell number in each sample relative to those in solvent-treated controls (set as 100%) (Figure 8).

We then assayed the four model compounds, lactacystin, polyoxin D, metronidazole, and paromomycin, to assess the applicability of this system for screening a chemical library. Lactacystin, metronidazole, and paromomycin treatment of the *in vitro E. histolytica* culture dose-dependently decreased the PI^−^ population (live trophozoites) while polyoxin D treatment did not; IC_50_ values of lactacystin, metronidazole and paromomycin were determined as 3.6 ± 0.14, 12.3 ± 2.47, and 11.5 ± 0.71 μM, respectively (Figure 8). The IC_50_ value of lactacystin treatment of trophozoites for 72 h or those of metronidazole or paromomycin treatment of trophozoites for 48 h were previously reported as ~1.0 (Makioka et al., 2002), 9.5 (Debnath et al., 2012; Penuliar et al., 2015) and ~12.6 μM (Penuliar et al., 2015), respectively. These values are close to those determined in the present study. The slightly higher IC_50_ values of lactacystin and metronidazole in the present study were probably because of the shorter incubation time (24 h). These results indicate that lactacystin, metronidazole, and paromomycin, all of which significantly inhibit cyst formation (see Figure 7A), also exert a cytotoxic effect on the proliferating trophozoites.

Taken together, the flow cytometry-integrated *Entamoeba* bioassay for cyst formation or trophozoite proliferation rapidly and quantitatively evaluated the effects of different compounds on the maintenance of the *Entamoeba* life cycle. Nevertheless, exploiting only the encystation assay may give misleading results; for instance, the present encystation assay (0–72 h) could not discriminate whether a compound acted on the mature cyst itself, on differentiating cells that appeared during the course of encystation, or only on trophozoites because there was a lag time to commit to trophozoite differentiation. To compensate for this drawback, two different 96-well plate-based *Entamoeba* bioassay and flow cytometry analysis systems were combined for the primary screening of a chemical library: one was for *E. invadens* encystation and the other was for *E. histolytica* trophozoite proliferation.

### Screening a chemical library to identify compounds that affect *Entamoeba* trophozoite proliferation and cyst formation

To validate the effectiveness of this methodology for the development of new drugs against amoebiasis, a chemical library was screened to obtain compounds that exert effect(s) on *Entamoeba* processes essential for maintenance of its life cycle: trophozoite proliferation, cyst formation, or both. The Pathogen Box provided by the MMV was chosen as a model chemical library, and contains 400 compounds exhibiting various scaffolds (https://www.pathogenbox.org/).

**Figure 9:**
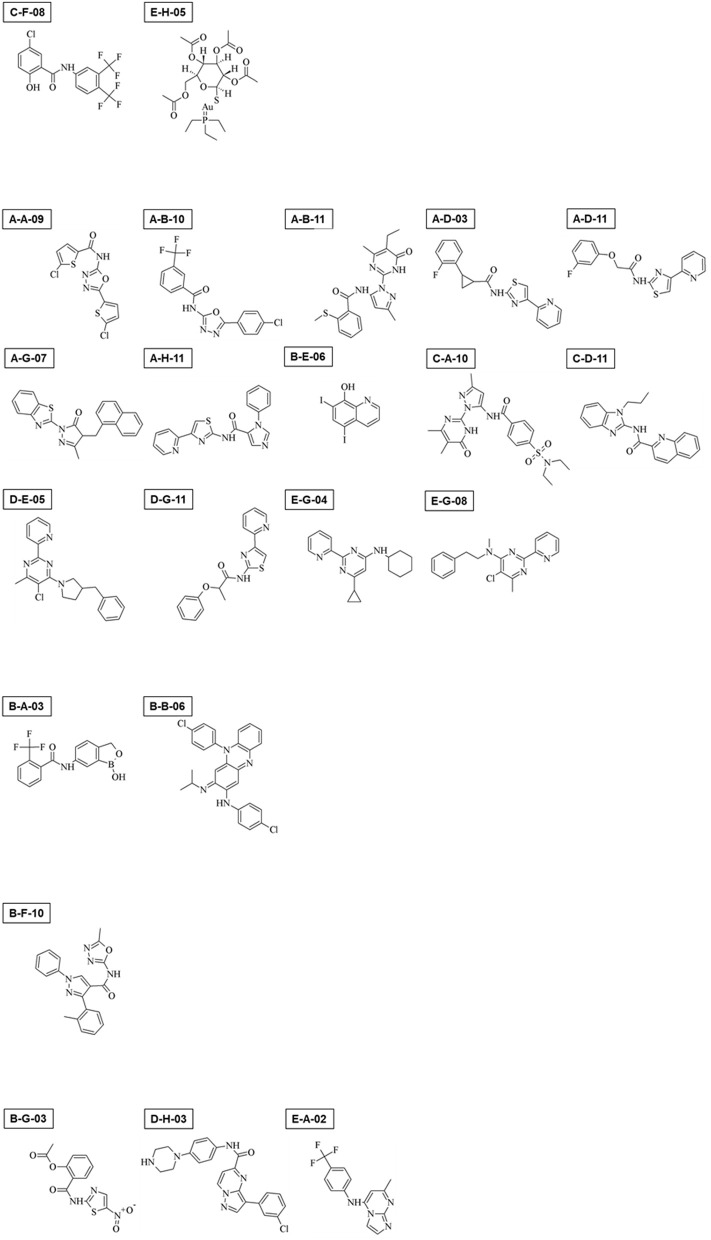
Chemical structures of 22 compounds that exerted significant effects on *Entamoeba* trophozoite proliferation, cyst formation, or both.

Among 400 compounds screened, 22 consistently showed a high negative effect at 10 μM (>80% reduction) in either cyst formation or the trophozoite proliferation assay, or both (Supplementary Figures S1A–F, Table 1; see Figure 9 for their structures); two compounds (C-F-08 and E-H-05) almost completely arrested both of these biological processes. Fourteen compounds (A-A-09, A-B-10, A-B-11, A-D-03, A-D-11, A-G-07, A-H-11, B-E-06, C-A-10, C-D-11, D-E-05, D-G-11, E-G-04, and E-G-08) almost completely halted cyst formation but only partially impaired trophozoite proliferation levels (35.0–74.9%). In contrast, two compounds (B-A-03 and B-B-06) almost completely arrested trophozoite proliferation but inhibited the cyst formation only partially (23.7–55.1%). Four compounds (B-F-10, B-G-03, D-H-03, and E-A-02) showed a biased inhibitory pattern; B-F-10 almost completely inhibited cyst formation, but did not affect trophozoite proliferation, whereas B-G-03, D-H-03, and E-A-02 showed the inverse effect.

**Table 1:** List of screened compounds that show significant effects on *Entamoeba* cyst formation and trophozoite proliferation.

			**Screening (encystation)**		**Screening (trophozoite proliferation)**	
**Rack**	**Position**	**Trivial Name**	**1st**	**2nd**	**1st**	**2nd**
			**Encystation rate (%)**		**Live trophozoite (%)**	
PlateC	F08		2.6	1.9	3.8	1.2
PlateE	H05	Auranofin	1.0	1.2	1.4	0.5
						
PlateA	A09		1.6	1.4	66.2	47.9
PlateA	B10		1.6	1.6	48.0	62.1
PlateA	B11		1.5	1.6	45.5	56.0
PlateA	D03		1.3	4.4	60.8	56.0
PlateA	D11		1.7	1.2	68.4	59.4
PlateA	G07		3.3	2.7	61.4	50.8
PlateA	H11		1.5	1.4	76.7	73.1
PlateB	E06	Iodoquinol	2.6	6.3	82.1	60.5
PlateC	A10		3.5	3.3	56.0	44.7
PlateC	D11		1.0	6.7	50.7	75.5
PlateD	E05		0.8	1.8	69.5	48.0
PlateD	G11		1.0	1.4	53.4	57.3
PlateE	G04		1.4	2.0	44.2	25.9
PlateE	G08		1.2	3.4	66.9	55.2
						
PlateB	A03		23.5	24.0	1.5	0.4
PlateB	B06	Clofazimine	31.9	78.3	3.5	0.9
						
PlateB	F10		1.7	6.3	102.3	94.2
PlateB	G03	Nitazoxanide	78.7	87.7	16.5	9.2
PlateD	H03		76.2	101.0	13.4	11.5
PlateE	A02		97.6	106.4	14.8	6.7

*Data from two independent cyst formation assays or trophozoite proliferation assays are summarized*.

**Figure 10:**
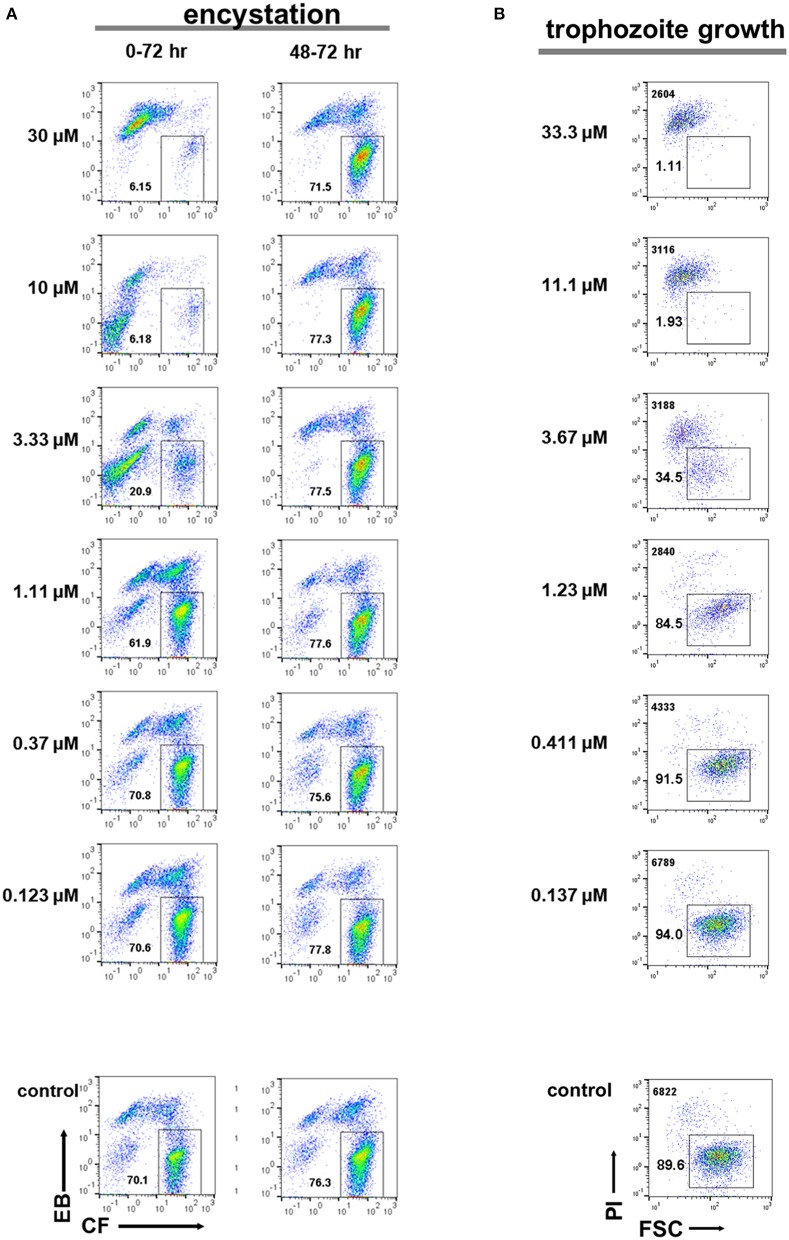
Effects of auranofin (E-H-05) on *E. histolytica* trophozoite proliferation and *E. invadens* cyst formation. **(A)** Encystation assay. Encystation-inducing culture was treated with auranofin immediately after induction (0–72 h). Encystation-inducing culture was treated with auranofin 48 h after induction. Flow cytometric analysis was performed 72 h after induction (48–72 h). **(B)** Trophozoite proliferation assay. The total cell number counted is indicated in the upper left corner. Representative data are shown from three experiments in **(A,B)**, respectively.

E-H-05 is auranofin, which is 10-times more potent than metronidazole against *E. histolytica* trophozoites and has been used as an FDA (food and drug administration in USA)-approved anti-rheumatoid drug (Debnath et al., 2012). IC_50_ values of auranofin for *Entamoeba* cyst formation and trophozoite proliferation were determined as 1.73 ± 0.70 and 0.690 ± 0.139 μM, respectively (Figures 10A,B; 0–72 h). Furthermore, addition of auranofin to the *Entamoeba* encystation-inducing culture at 48 h post induction did not affect the number of cysts formed at 72 hr, similar to the effect of metronidazole (Figure 10A; see Figure 7B). These results can be interpreted as an indirect negative effect of auranofin on *Entamoeba* cyst formation by causing *Entamoeba* cell dysfunction; the cell population includes proliferating trophozoites and differentiating cells that do not yet show characteristics of mature cysts (see Figures 4, 5; 48 h).

Hence, combining the two different 96-well plate-based *Entamoeba* bioassay and flow cytometry analysis systems (cyst formation and trophozoite proliferation) is effective for the development of new drugs against amoebiasis, such as anti-amoebic and amoebiasis transmission-blocking drugs.

## Discussion

We have developed two standardized 96-well plate-based *Entamoeba* bioassay and flow cytometry systems: one is for an encystation assay using *E. invadens* and the other is for a trophozoite proliferation assay using *E. histolytica*. Each system can rapidly, reproducibly and quantitatively analyze the biological processes essential for maintenance of the *Entamoeba* life cycle; therefore, studies exploiting each or both systems will enable investigations from both biological and medical perspectives. For example, visualization of the *Entamoeba* encystation cell differentiation process was achieved by identifying and characterizing distinct cell populations. The feasibility of providing potential leads for the development of amoebiasis transmission-blocking and anti-amoebic drugs was demonstrated by screening a chemical library, the Pathogen Box from MMV (https://www.pathogenbox.org/).

Recently, Welter et al. reported using flow cytometry for fixed *E. invadens* prepared from an encystation-inducing culture with the fluorescent dye, Congo Red. This method can separate the trophozoite and cyst populations and assess the effect of compounds on the dynamics of these two populations (Welter et al., 2017). In the present study, as well as a fluorescent dye (CF) that stains chitin (Arroyo-Begovich et al., 1980), a dye (EB) that stains membrane (Hed et al., 1983) were used and more time points during encystation were then analyzed to monitor the dynamics of population changes. A time course study using the present system for *E. invadens* encystation combined with fluorescence microscopy revealed that differentiation of proliferating trophozoites into dormant, mature cysts involves at least two distinct cell types that exist as sequential precursors during encystation. One is morphologically similar to the proliferating trophozoite but appears less motile, and emits EB fluorescence at the same level as proliferating trophozoites. The other is a round cell that looks like the mature cyst, and emits CF and EB fluorescence at the same levels as the mature cyst and the proliferating trophozoite, respectively. Detecting these two cell types, each of which shows mixed characteristics of proliferating trophozoites and mature cysts, is intriguing because it provides strong evidence for the existence of transient forms during encystation, as has been suggested (Chatterjee et al., 2009). However, correlation of the identified precursor cells to the assumed transient forms remains undetermined. In addition, more detailed characterization of the two precursor cell types is needed at cellular and molecular levels, to provide new mechanistic insights into *Entamoeba* encystation.

Other intriguing cells include the CF^−^/EB^−^, CF^low^/EB^strong^, and CF^+^/EB^strong^ populations. The CF^−^/EB^−^ and CF^low^/EB^strong^ cells produced CF-signal at lower levels, suggesting that these cells don't synthesize or accumulate normal levels of chitin, the target of the CF dye. Meanwhile, the CF^low^/EB^strong^ and CF^+^/EB^strong^ cells gave a much stronger EB-signal, indicating that these cells abnormally accumulate EB dye. Collectively, these findings indicate that the CF^−^/EB^−^, CF^low^/EB^strong^, and CF^+^/EB^strong^ populations are not normal forms, and that they appear to fail in the differentiation process of *Entamoeba* encystation. Based on the findings from the time course study, we propose a scenario for the cell differentiation process of *Entamoeba* encystation (Figures 6A,B).

The precise mechanism by which *Entamoeba* cells are stained with EB dye remains to be elucidated. The mechanism that damaged cells and disrupted blood vascular system or blood brain barrier are exclusively permeable to EB is widely recognized and is a basis for various mammalian biological studies, such as measurement of polymorphonuclear leukocyte infiltration (Griswold et al., 1989; Senaldi et al., 1994; Saunders et al., 2015). In *Entamoeba* study, however, it is unlikely because time course analysis of *Entamoeba* encystation indicates that EB^+^ cells sequentially become EB^−^ cells, indicating that EB^+^ cells are neither dead nor dying. It is also unlikely that EB binds to serum albumin (SA) that is associated with *Entamoeba* cells because, despite cultivating *Entamoeba* cells in the presence of SA, the cells were washed several times with PBS before treating with EB, and SA is not internalized into *Entamoeba* cells. However, the presence of an SA-binding protein and/or an SA-like protein in *Entamoeba* cannot be ruled out. The most plausible mechanism is for EB to stain the membrane of *Entamoeba* cells, similarly to its action on human neutrophils (Hed et al., 1983). Molecular identification of the target to which EB binds and unraveling the molecular and cellular mechanisms underlying EB fluorescence changes during encystation will inform new topics on this differentiation process.

This study and that of Welter et al. (2017) for determining the effects of compounds on encystation by quantifying *E. invadens* cyst formation are essentially the same; therefore, the usefulness of flow cytometry to screen compounds that show a significant effect on *Entamoeba* encystation was confirmed by two distinct procedures. However, as is evident from the present study, an important issue to be addressed is whether each compound directly affects the encystation process, or has an indirect effect by damaging trophozoites. As a clue to solve this issue, the present study shows that compounds that halt *Entamoeba* cyst formation do not necessarily act on the mature cyst; for example, metronidazole and auranofin did not exert their effects on differentiated *Entamoeba* cells that possess similar characteristics to mature cysts. This result, however, does not provide an answer to the question of whether trophozoites themselves or differentiating cells that appeared during the course of encystation are targeted by the above compounds.

As a solution, in this study, we standardized a flow cytometry method to exclusively analyze proliferating *E. histolytica* trophozoites and combined it with that for analyzing cyst formation in *E. invadens*. The effectiveness of this combined system was demonstrated by providing very similar IC_50_ values for lactacystin, metronidazole, and paromomycin for cyst formation and trophozoite proliferation. This finding indicates compounds that halt cyst formation by causing trophozoite dysfunction, and that the molecules targeted by them have fundamental roles in the *Entamoeba* life cycle; lactacystin and paromomycin impair the ubiquitin proteasome system and protein synthesis, respectively, and metronidazole induces damage of DNA or proteins (Liu and Weller, 1996; Makioka et al., 2002; Penuliar et al., 2015; Mi-ichi et al., 2016; Prokhorova et al., 2017). In addition, two compounds, which almost completely arrested both cyst formation and trophozoite proliferation, were identified using the combined system to screen the chemical library, the Pathogen Box of MMV (https://www.pathogenbox.org/). Importantly, one of these two compounds, auranofin was previously suggested to exert a cytotoxic effect on *E. histolytica* trophozoites by enhancing reactive oxygen-mediated cell killing via inhibition of thioredoxin reductase, an enzyme critical for preventing reactive oxygen generation (Debnath et al., 2012). However, the possibility still remains that cells undergoing differentiation from proliferative trophozoites are sensitive to compounds such as metronidazole and auranofin. Therefore, discrimination of these cells regarding drug sensitivity and elucidating the underlying molecular mechanisms present not only new topics in *Entamoeba* encystation research but also important issues to be addressed for the development of new drugs against amoebiasis.

Interestingly, the combined system-based screening of the chemical library also identified four compounds that show an effect on either cyst formation (one compound) or trophozoite proliferation (three compounds). This finding is intriguing because the molecules targeted by these compounds are suggested to be stage-specifically expressed to exert an essential role in distinct stages of the *Entamoeba* life cycle: the proliferative trophozoite or dormant cyst stages. In addition, 16 compounds were identified that almost completely abolished either cyst formation or trophozoite proliferation, but inhibited the other process only partially. This finding indicates that the target molecules of these 16 compounds also play important roles in the both trophozoite and cyst stages, but that their expression levels differ in each stage. However, the possibility cannot be ruled out that distinct levels of inhibition by the 20 compounds are attributed to the diversity of their target molecules between *E. histolytica* and *E. invadens*; for the above case of one compound that inhibited cyst formation, its target molecule may exist only in *E. invadens*, and not in *E. histolytica*. Hence, identifying and characterizing the molecules targeted by the screened compounds will provide new mechanistic insight for *Entamoeba* encystation, a cell differentiation process.

The effectiveness of the combined system of the *E. invadens* cyst formation and *E. histolytica* trophozoites proliferation assays is also demonstrated for applied perspective. This combined system identified twenty-two compounds that showed significant, negative effects on *Entamoeba* cyst formation, trophozoite proliferation, or both by screening 400 compounds with diverse scaffolds, the Pathogen Box from MMV (https://www.pathogenbox.org/). All in all, auranofin (which is identified as E-H-05 in the Pathogen Box) is a promising lead for the development of new anti-amoebic drugs because of almost completely arresting the both biological processes. The argument is essentially the same with that of Debnath et al. (2012), in which the different assay system was exploited. In view of inhibitory profile observed in the present assay system, C-F-08 may also be a promising lead compound because of its similarity to auranofin. Among 14 compounds that almost completely halted cyst formation but only partially impaired trophozoite proliferation, iodoquinol (which is identified as B-E-06 in the Pathogen Box) was included. This is used as a luminal amoebicide and recommended to administer asymptomatic cyst carriers (Levine, 1991; Marie and Petri, 2013). Considering the similarity observed in their inhibitory profiles, the rest 13 compounds also be a potential lead for the development of new amoebiasis transmission-blocking drugs. However, determination of cells affected by the each compound is needed; for instance, trophozoite, differentiating cells appeared during encystation, and/or cyst. B-F-10 may be the same type with the above 14 compounds, but, as discussed in the previous paragraph, we should be aware that its target may exist only in *E. invadens*, and not in *E. histolytica*. Among other five compounds that exert more severe effect on trophozoite proliferation than cyst formation, nitazoxamide (which is identified as B-G-03 in the Pathogen Box) was included. Nitazoxamide is proposed to be a noncompetitive inhibitor of pyruvate:ferredoxin oxidoreductase, a critical enzyme in the main route for ATP supply in *E. histolytica* (Hoffman et al., 2007); therefore, it is plausible that nitazoxamide-susceptible cell requires pyruvate:ferredoxin oxidoreductase activity to fulfill high ATP demand, such as proliferating *E. histolytica* trophozoite. Furthermore, all 22 compounds screened exhibit bactericidal as well as parasiticidal activities (Table 2); therefore, the success of this new trial confirms that the Pathogen Box is an appropriate resource for the development of new drugs against a wide range of human pathogens. Importantly, it also confirms relevance of the presented combined system, in which the threshold is set as >80% reduction by 10 μM compound, for a primary screen of a chemical library to provide potential leads for the development of new anti-amoebic and amoebiasis transmission-blocking drugs.

**Table 2:** Summary of the 22 screened compounds: specificities against various pathogens.

**Rack**	**Position**	**Compound ID**	**Trivial name**	**Target infectious diseases^#^**
PlateC	F08	MMV687807		Tuberculosis, Candidiasis (Vila and Lopez-Ribot, 2017), and Toxoplasmosis (Spalenka et al., 2018)
PlateE	H05	MMV688978	Auranofin	Amoebiasis and Rheumatoid arthritis
PlateA	A09	MMV676501		Tuberculosis
PlateA	B10	MMV102872		Tuberculosis
PlateA	B11	MMV676477		Tuberculosis
PlateA	D03	MMV1028806		Malaria
PlateA	D11	MMV676409		Tuberculosis
PlateA	G07	MMV676558		Tuberculosis
PlateA	H11	MMV676512		Tuberculosis
PlateB	E06	MMV002817	Iodoquinol	Amoebiasis (Marie and Petri, 2013) and Onchocerciasis
PlateC	A10	MMV595321		Trypanosomiasis and Leishmaniasis
PlateC	D11	MMV1030799		Malaria
PlateD	E05	MMV659004		Trypanosomiasis and Leishmaniasis
PlateD	G11	MMV676411		Tuberculosis
PlateE	G04	MMV021013		Tuberculosis
PlateE	G08	MMV658988		Trypanosomiasis and Leishmaniasis
PlateB	A03	MMV652003		Trypanosomiasis and Leishmaniasis
PlateB	B06	MMV687800	Clofazimine	Leprosy and Visceral Leishmaniasis
PlateB	F10	MMV020320		Malaria
PlateB	G03	MMV688991	Nitazoxanide	Amoebiasis (Marie and Petri, 2013) and Cryptosporidiosis
PlateD	H03	MMV022478		Malaria
PlateE	A02	MMV011229		Malaria

*Compound IDs are taken from Medicine for Malaria Venture (MMV; https://www.pathogenbox.org/)*.

# *Information was obtained from MMV and references*.

Meanwhile, this system can also be applied to vaccine development; it can be used to screen antibodies that inhibit the *Entamoeba* trophozoite proliferation, cyst formation, or both, which will lead to the development of anti-amoebic and amoebiasis transmission-blocking vaccines. Attainment of the ultimate goals—developing new drugs and vaccines against amoebiasis—by exploiting the methodology demonstrated in this study is urgently needed in amoebiasis medicine because of limited available clinical options. Contribution of the demonstrated methodology to this important medical issue will become more substantial, if an automated, high-throughput system can be integrated with it, such as a 384-well system.

In conclusion, we present a methodology that can be widely adopted for *Entamoeba* study. Developing methodologies, such as that described here, is still required for various areas of the *Entamoeba* field.

## Author contributions

FM designed the experiments and analyzed the data. FM, YM, and VT performed the experiments. FM, YM, VT, and HY interpreted the data. FM and HY wrote the manuscript.

### Conflict of interest statement

The authors declare that the research was conducted in the absence of any commercial or financial relationships that could be construed as a potential conflict of interest.

## Supplementary material

Supplementary Figure S1 Secreening 400 compounds with diverse scaffolds from the Pathogen Box of MMV. **(A–E)** The effect(s) on *Entamoeba* cyst formation (upper) or trophozoite proliferation (lower) of 80 compounds from the Pathogen Box in plates **(A–E)**, respectively. In the trophozoite proliferation assay, the total cell number counted is also indicated. Representative data are shown from two independent experiments. **(F)** Controls of cyst formation (upper) and trophozoite proliferation (lower) assays. Average percentages of mature cysts and average cell numbers of live trophozoites were 70.2 ± 0.21% and 6824 ± 324 (*n* = 3), respectively. These data were obtained from each assay and used as controls at 100% to calculate the inhibition rate of each compound tested.
