# Supplementary material for: A Flow Cytometry Method for Dissecting the Cell Differentiation Process of Entamoeba Encystation
Source: Front Cell Infect Microbiol. 2018 Jul 24;8:250. doi: 10.3389/fcimb.2018.00250 (PMC6066566; doi:10.3389/fcimb.2018.00250)

Supplementary Figure S1

(A)

cyst formation

trophozoite proliferation

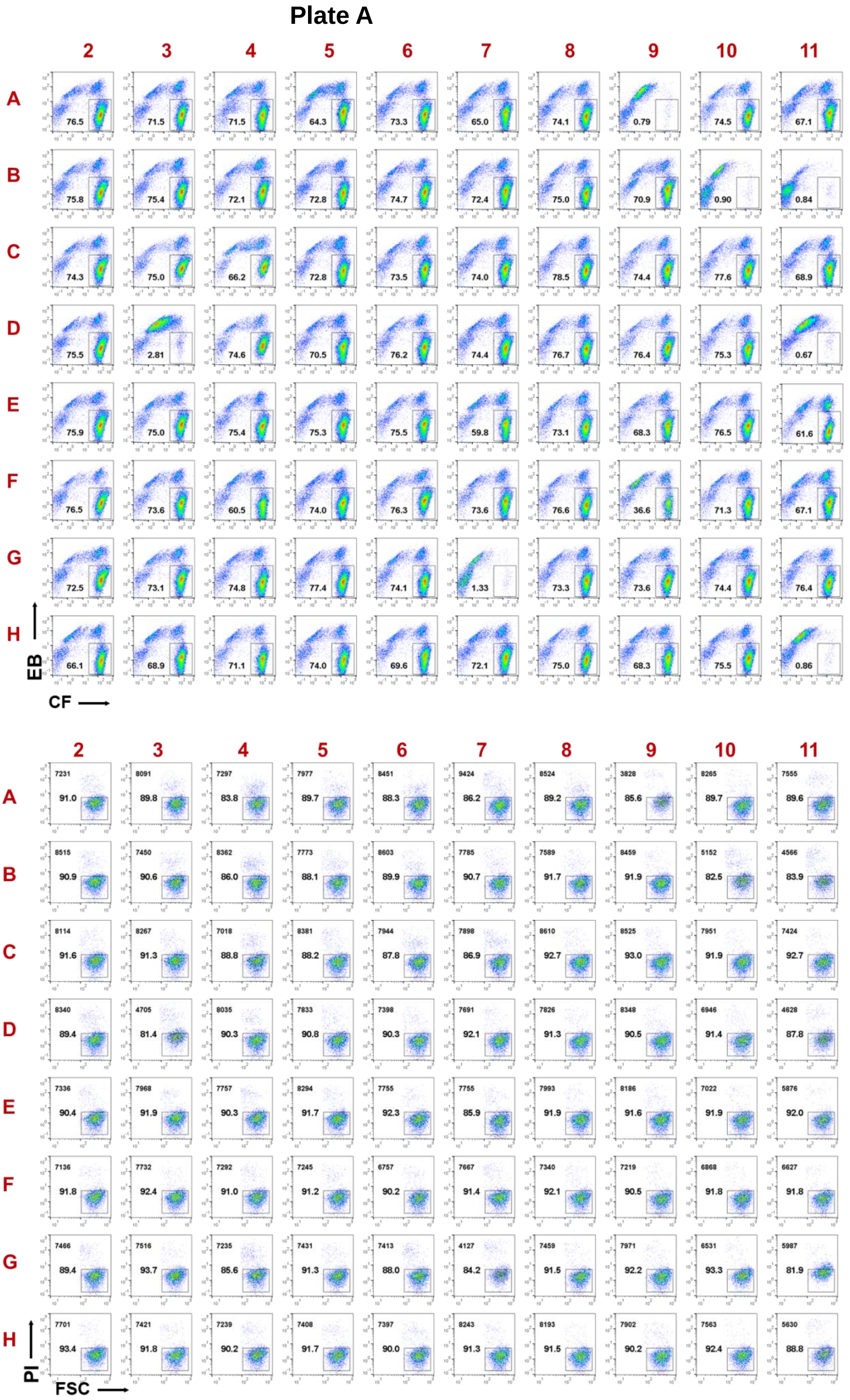

(B)

Plate B

cyst formation

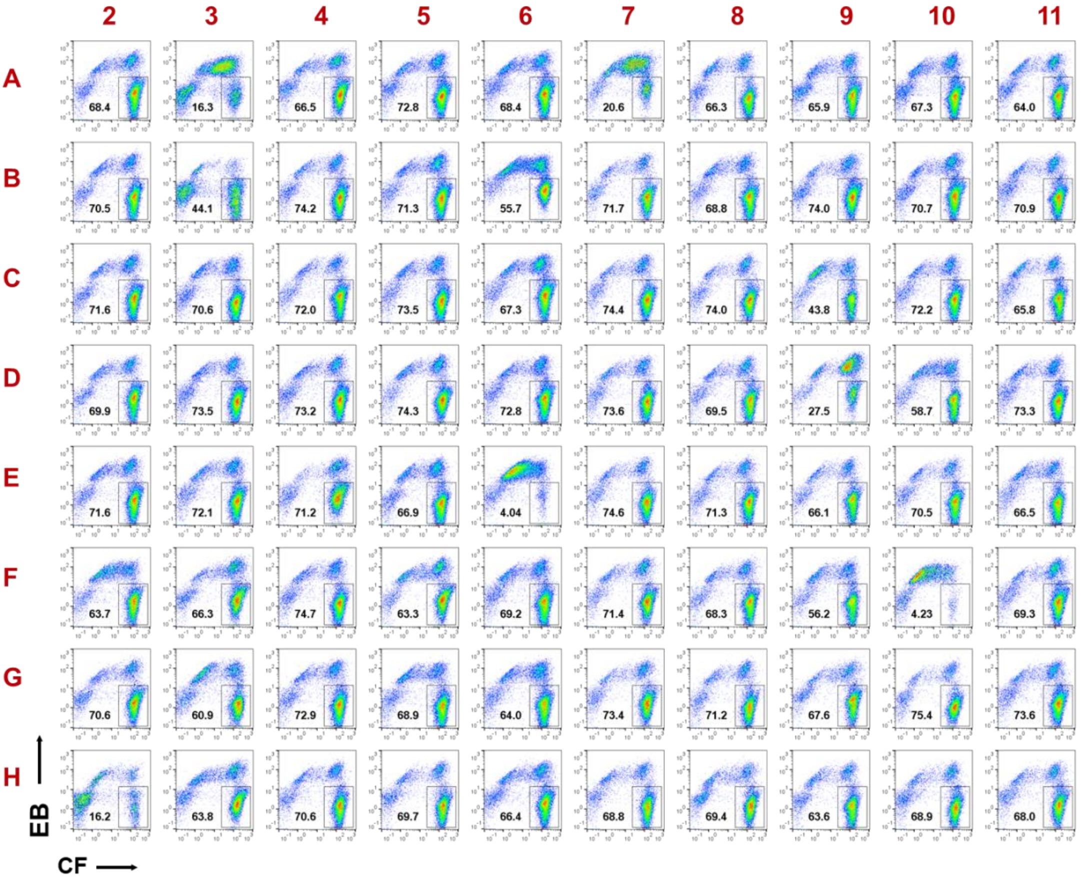

trophozoite proliferation

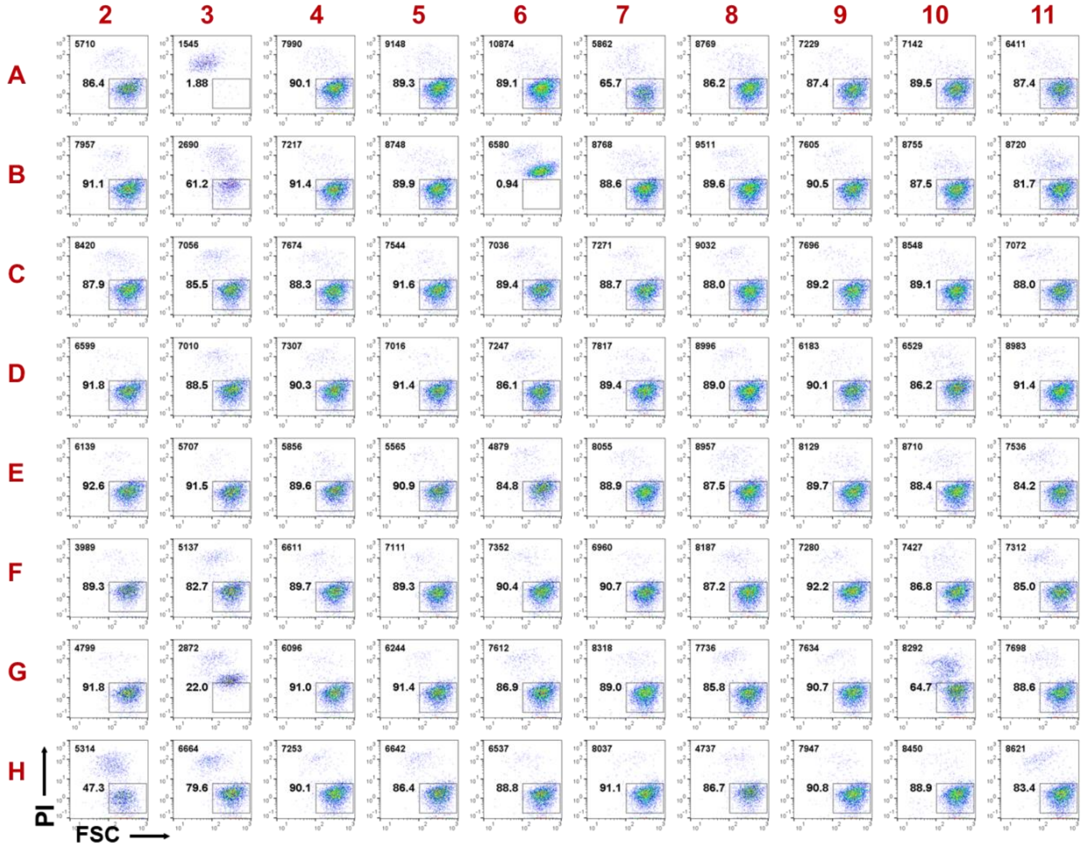

(C)

Plate C

cyst formation

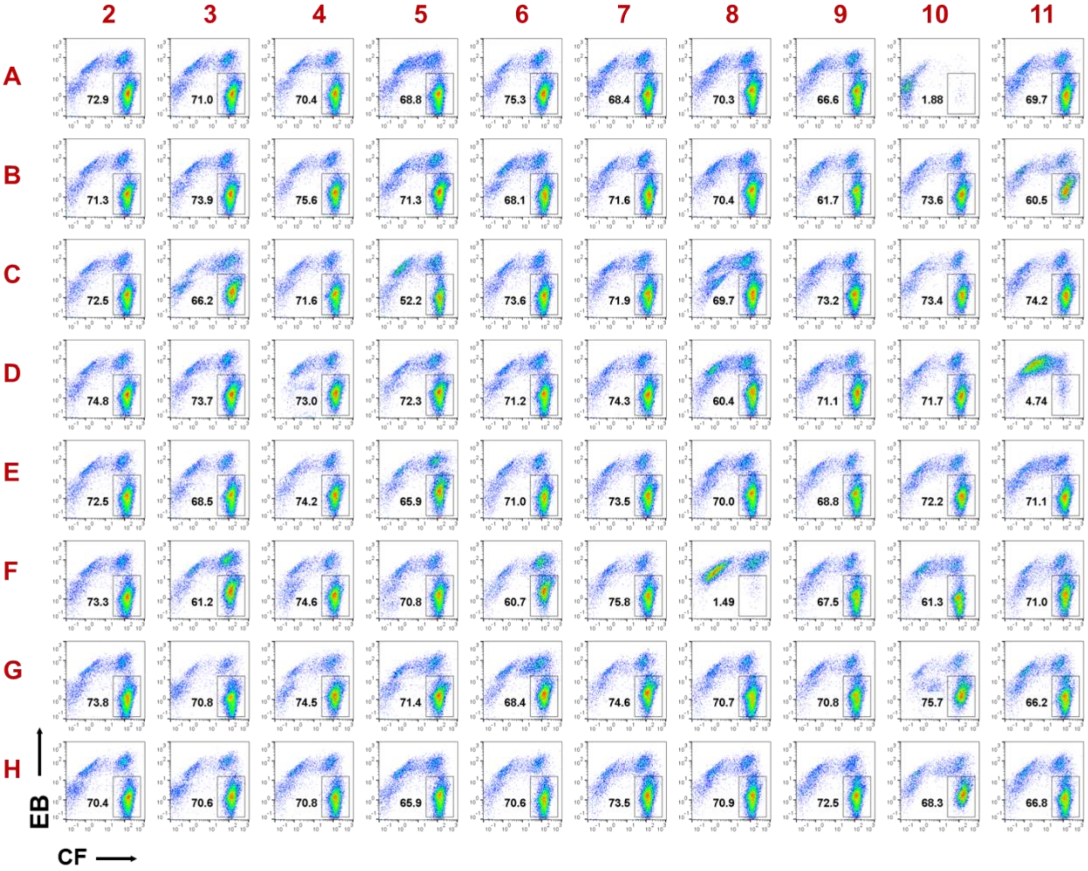

trophozoite proliferation

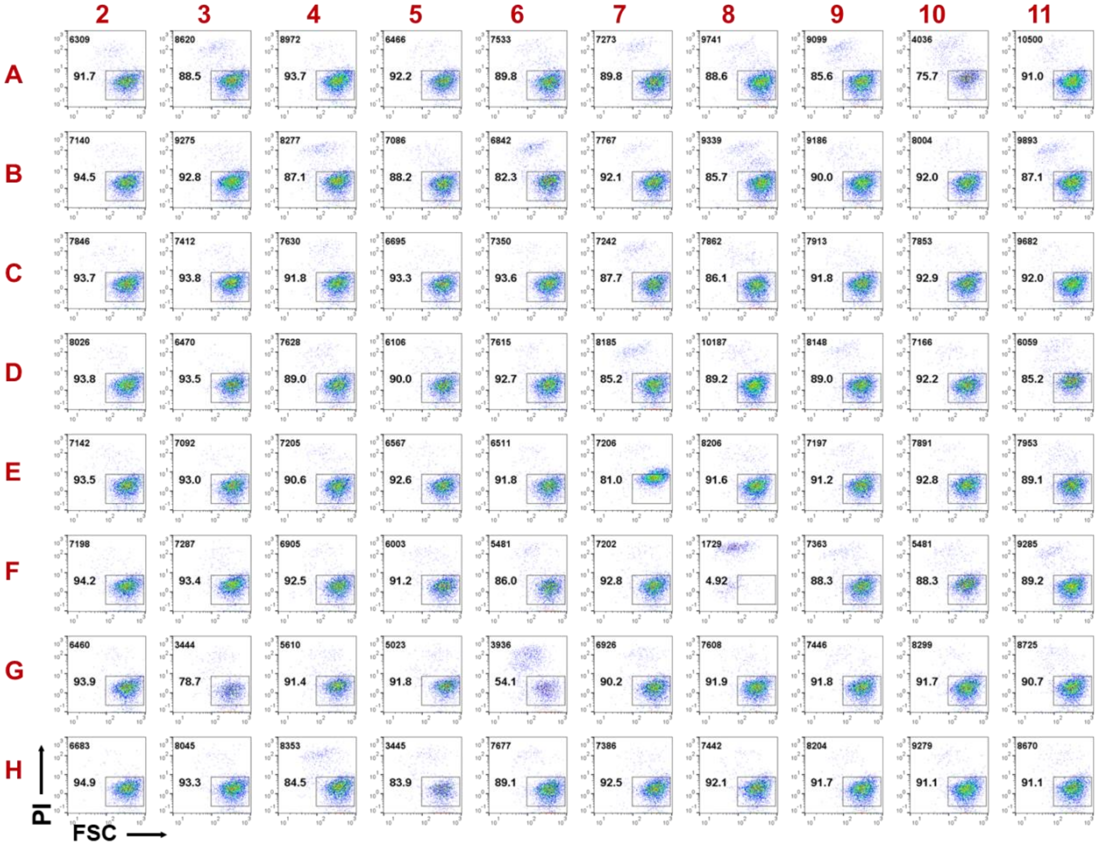

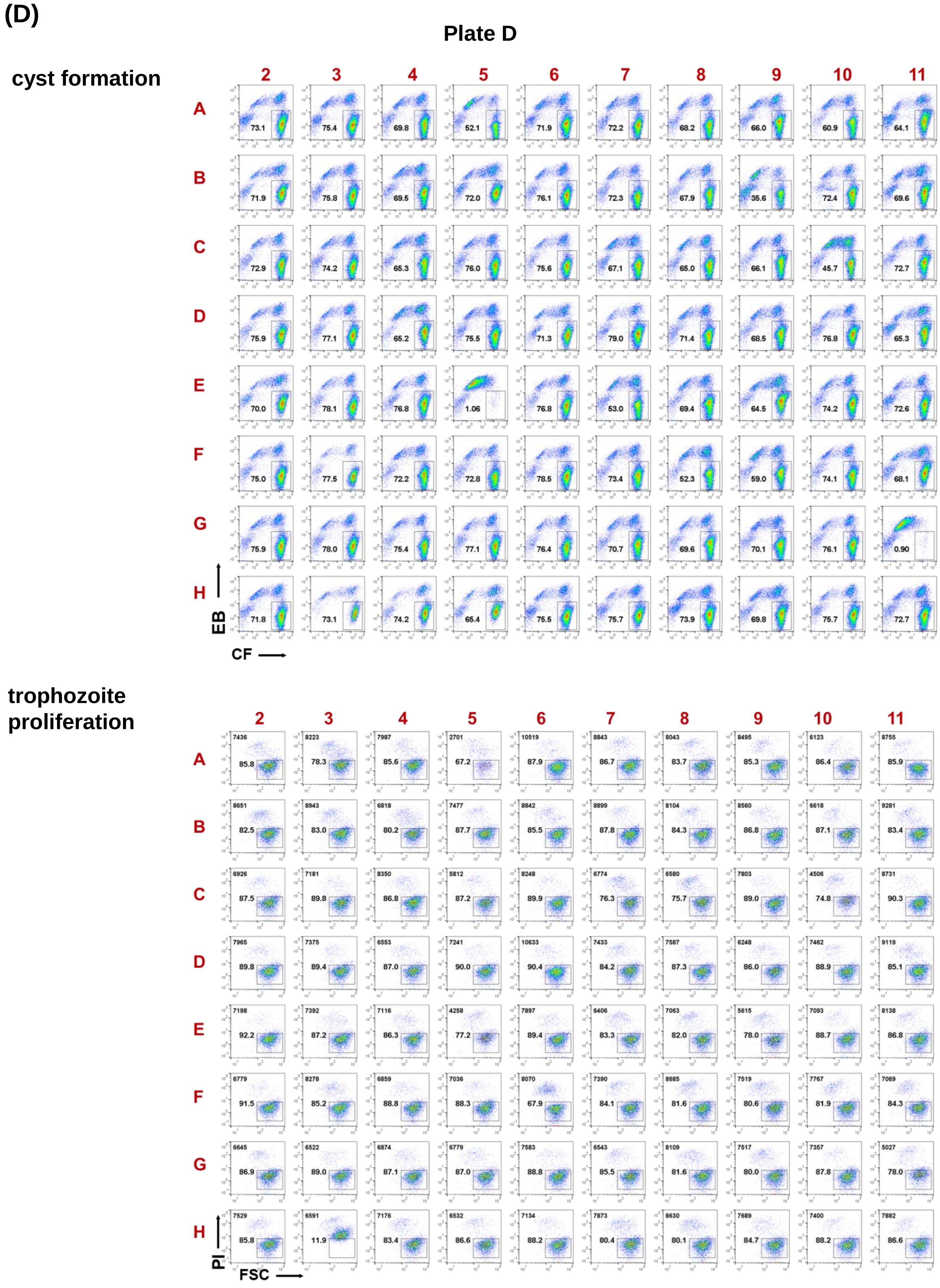
(D)
Plate D
cyst formation
2 3 4 5 6 7 8 9 10 11
A B C D E F G H
EB
CF
trophozoite proliferation
2 3 4 5 6 7 8 9 10 11
A B C D E F G H
PI
FSC

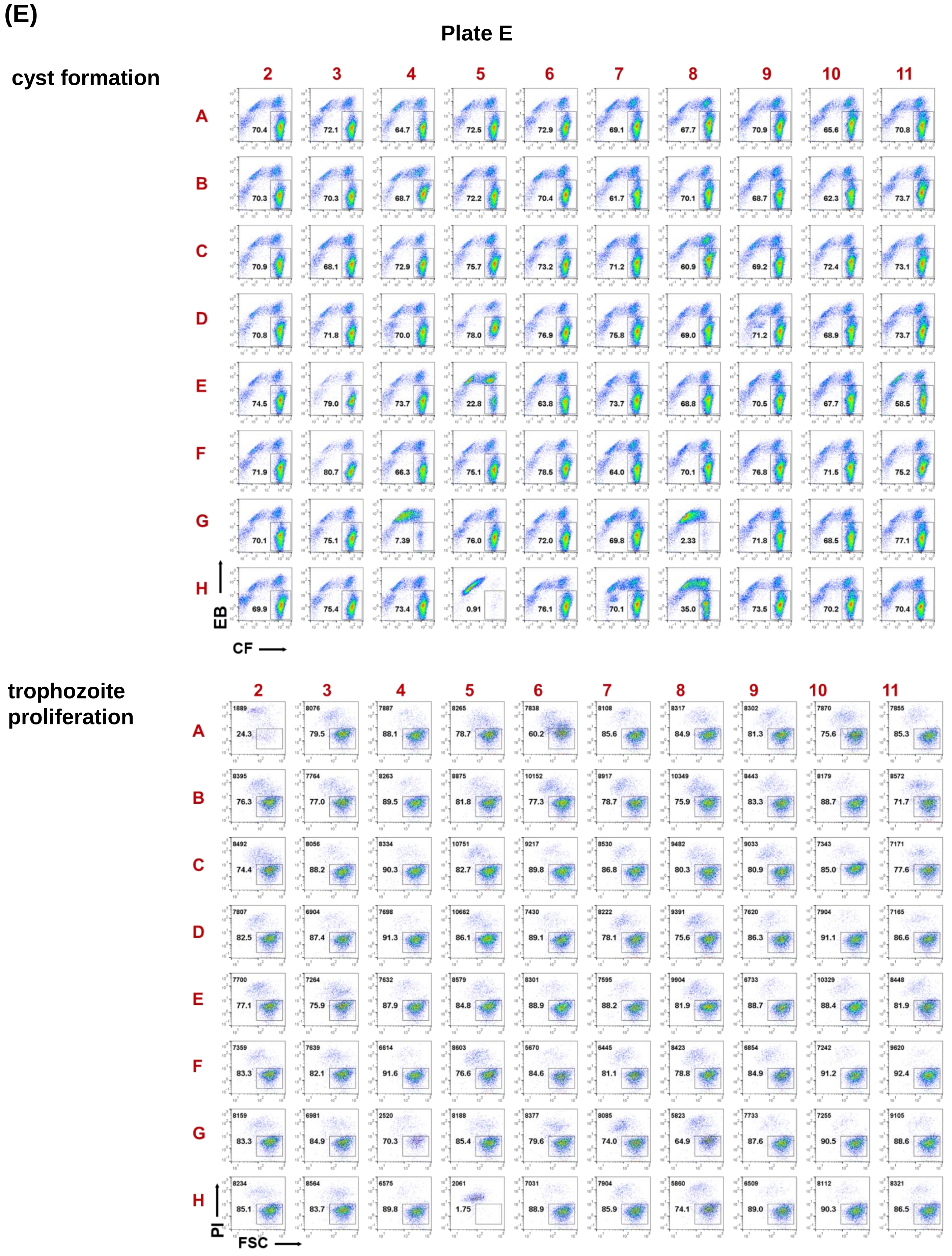
(E)
Plate E
cyst formation
2 3 4 5 6 7 8 9 10 11
A 70.4 72.1 64.7 72.5 72.9 69.1 67.7 70.9 65.6 70.8
B 70.3 70.3 68.7 72.2 70.4 61.7 70.1 68.7 62.3 73.7
C 70.9 68.1 72.9 75.7 73.2 71.2 60.9 69.2 72.4 73.1
D 70.8 71.8 70.0 78.0 76.9 75.8 69.0 71.2 68.9 73.7
E 74.5 79.0 73.7 22.8 63.8 73.7 68.8 70.5 67.7 58.5
F 71.9 80.7 66.3 75.1 78.5 64.0 70.1 76.8 71.5 75.2
G 70.1 75.1 7.39 76.0 72.0 69.8 2.33 71.8 68.5 77.1
H 69.9 75.4 73.4 0.91 76.1 70.1 35.0 73.5 70.2 70.4
EB
CF
trophozoite proliferation
2 3 4 5 6 7 8 9 10 11
A 1889 24.3 8076 79.5 7887 88.1 8265 78.7 7838 60.2 8108 85.6 8317 84.9 8302 81.3 7870 75.6 7855 85.3
B 8395 76.3 7764 77.0 8263 89.5 8875 81.8 10152 77.3 8917 78.7 10349 75.9 8443 83.3 8179 88.7 8572 71.7
C 8492 74.4 8056 88.2 8334 90.3 10751 82.7 9217 89.8 8530 86.8 9482 80.3 9033 80.9 7343 85.0 7171 77.6
D 7807 82.5 6904 87.4 7698 91.3 10662 86.1 7430 89.1 8222 78.1 9391 75.6 7620 86.3 7904 91.1 7165 86.6
E 7700 77.1 7264 75.9 7632 87.9 8579 84.8 8301 88.9 7595 88.2 9904 81.9 6733 88.7 10329 88.4 8448 81.9
F 7359 83.3 7639 82.1 6614 91.6 8603 76.6 5670 84.6 6445 81.1 8423 78.8 6854 84.9 7242 91.2 9620 92.4
G 8159 83.3 6981 84.9 2520 70.3 8188 85.4 8377 79.6 8085 74.0 5823 64.9 7733 87.6 7255 90.5 9105 88.6
H 8234 85.1 8564 83.7 6575 89.8 2061 1.75 7031 88.9 7904 85.9 5860 74.1 6509 89.0 8112 90.3 8321 86.5
PI
FSC

(F)

controls

cyst formation

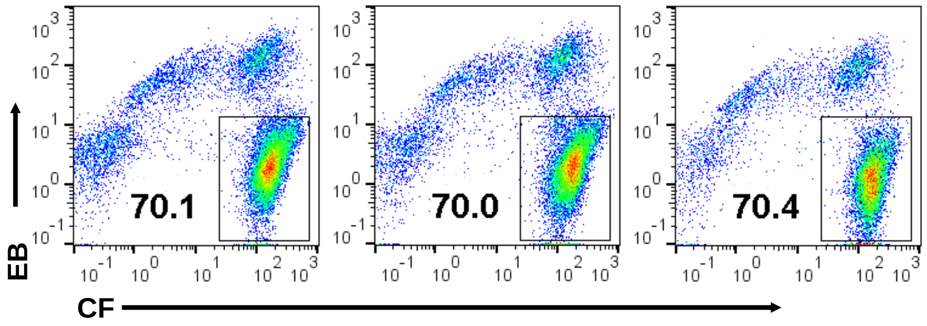

trophozoite proliferation

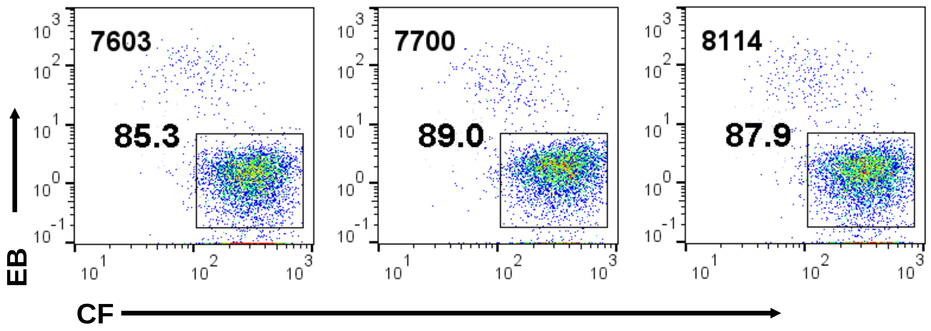

Supplement: Supplementary Figure S1 — Secreening 400 compounds with diverse scaffolds from the Pathogen Box of MMV. (A–E) The effect(s) on Entamoeba cyst formation (upper) or trophozoite proliferation (lower) of 80 compounds from the Pathogen Box in plates (A–E), respectively. In the trophozoite proliferation assay, the total cell number counted is also indicated. Representative data are shown from two independent experiments. (F) Controls of cyst formation (upper) and trophozoite proliferation (lower) assays. Average percentages of mature cysts and average cell numbers of live trophozoites were 70.2 ± 0.21% and 6824 ± 324 (n = 3), respectively. These data were obtained from each assay and used as controls at 100% to calculate the inhibition rate of each compound tested. [file Image_1.PDF]
